# Service-Learning as a Practical Introduction to Undergraduate Public Health: Benefits for Student Outcomes and Accreditation

**DOI:** 10.3389/fpubh.2019.00063

**Published:** 2019-04-02

**Authors:** Meghan R. Mason, Elizabeth Dunens

**Affiliations:** ^1^Public Health Department, St. Catherine University, St. Paul, MN, United States; ^2^The Center for Community Work and Learning, St. Catherine University, St. Paul, MN, United States

**Keywords:** undergraduate, public health, service-learning, student learning outcomes, accreditation

## Abstract

Since the mid-1980s, service-learning has gained recognition as a pedagogical model in higher education with exciting potential for students' academic, civic, and professional development (1). Deemed a high-impact educational practice by the American Association of Colleges and Universities (AAC&U), extant research points to student learning, engagement, and retention benefits from community-based experiences integrated into curriculum (2, 3). Numerous studies have examined best practices for service-learning from varying stakeholder perspectives (faculty, student, and community partner) and disciplines, however, due to the recent development of public health as a major offering in U.S. undergraduate education, the value of service-learning within the discipline should be further explored. While recommendations for service-learning in undergraduate public health programs have been provided, no evaluation of the impact on student learning outcomes has been conducted (4). This study presents one university's model of service-learning in introductory public health courses, and results from the analysis of two datasets representing students' experience with service-learning in undergraduate public health curriculum. Findings provide empirical support of the effectiveness of this pedagogy for advancing student learning and the achievement of foundational accreditation domains outlined by the Council on Education for Public Health (CEPH).

## Introduction

The start of undergraduate public health education in the United States can be traced back to 2003 when the American Schools of Public Health (now American Schools and Programs of Public Health) launched a taskforce for undergraduate education (5). This taskforce was a response to an Institute of Medicine report that recommended all undergraduates, regardless of their discipline, have access to public health education (6). Interest in public health grew, and the development of majors followed. In 2002, there were 1,438 degree conferrals at the undergraduate level for Classification of Instruction (CIP) codes in the area of public health, increasing 5-fold to 6,464 by 2012 (7) and to 12,895 conferrals in 2016 (8). While monitoring and accreditation of graduate programs has been in place since 1946 (6), it was not until 2003 (9) that the Council for Education in Public Health (CEPH) first accredited undergraduate programs under already-accredited graduate schools of public health. Only recently, in 2013, has CEPH opened accreditation to stand-alone undergraduate programs (9). As demand for undergraduate majors in public health has increased, so too has interest from colleges and universities in seeking accreditation.

St. Catherine University's undergraduate public health major was founded in 2009 and is currently seeking accreditation under the CEPH 2016 criteria (10). St. Catherine's is a private, Catholic, liberal arts university located in St. Paul, Minnesota with 4,724 students enrolled across associate, undergraduate, and graduate degree programs. The undergraduate degree in public health is the first of its kind in Minnesota and includes four concentrations: Health Sciences, Public Policy, Health Education, and Community Health Worker. In <10 years, this degree has become the second most popular major for St. Catherine undergraduates. It is also one of the most diverse majors when it comes to student identities represented. Given the social justice orientation of the University and the community-based nature of the discipline, the department chose to integrate service-learning into the undergraduate public health curriculum in fall of 2015. Inclusion of community engagement in the first course that majors encounter (Foundations in Public Health) also addresses two of the CEPH 2016 accreditation criteria: (1) Students should have learning experiences that address the “socioeconomic, behavioral, biological, environmental, and other factors that impact human health and contribute to health disparities” (Foundational Domains); and (2) Students are required to have experiential learning activities that support their didactic education (Cumulative and Experiential Activities). In this paper we analyze student data collected since the inception of the service-learning course to examine the effectiveness of using this pedagogical model for introductory, undergraduate public health coursework.

## Service-Learning

Service-learning is an experiential learning model involving the incorporation of community engagement into course curriculum to enhance student learning outcomes and advance community efforts. Service-learning is not simply volunteer activity tied to a course, but an intentional pedagogy that “ensures that both the service enhances the learning and the learning enhances the service” [(11), p. 12]. In order to achieve this, courses should be grounded in reciprocity, reflection, and social change. At St. Catherine University, faculty work with staff from the university's Center for Community Work and Learning (CWL) to identify student engagement opportunities at community organizations with which CWL has an ongoing relationship. Faculty, community partners and CWL staff co-design service-learning on a course-by-course basis to meet learning objectives and the expressed needs of the community partner. This focus on relationship is informed by community engagement theory and praxis, and an aspiration to achieve “transformational” over “transactional” collaborations (12). The scaffolding of in-class and assignment-based reflection activities before, during and after service-learning is essential to students “mak[ing] meaning of their experiences in and with communities and enhancing the quality of thinking, of learning, of service, and of partnerships” (13). This intentional bridging of course and community learning also prevents the phenomenon of students perceiving their engagement as an unnecessary “add on” to the course (14, 15).

Regarding social change, in our service-learning experience we see this happen in two ways. First, our students are contributing to and allying with local, justice-oriented community efforts. Secondly, through engagement in critical service-learning, our students advance toward becoming public health practitioners who understand “the systemic and institutionalized nature of oppression [and]…personal and institutional contributions to social problems and measures that may lead to social change” [(16), p. 54]. Developing students' social justice literacy aligns with institutional values and prepares them to be effective public health practitioners. As undergraduate public health programs continue to emerge and evolve, we present one model that has worked in our Foundations of Public Health class to meet student, community, and accreditation outcomes.

## Foundations in Public Health

St. Catherine's Foundations of Public Health course is intended to introduce students to the population perspective and ecological nature of public health. Additionally, students examine how health is distributed unequally across race, gender, class, and environment, with a focus on health disparities in our own communities. Broadly, the learning objectives for the Foundations of Public Health course fall under three primary themes: (1) foundational concepts of health, public health, and health systems; (2) social justice implications of public health; (3) beginning level professional attitudes, behavior, and communication. These themes provide students with the opportunity to explore public health as a major while reflecting on and integrating their learning with the broader St. Catherine University liberal arts and Catholic social teaching traditions.

To tie the three primary learning themes together, the Foundations of Public Health course has a cumulative Community Health Assessment project that includes the following components: (1) community definition and description; (2) quantitative data and statistics; (3) walking survey; (4) key informant interviews; (5) reflection on service-learning; (6) opportunities for improving the health of the community (Appendix A). This project is grounded in students' service-learning experience. For service-learning, students engage in small teams at a non-profit organization on three occasions for a total of 6 h of community-based work. Service-learning activities range from working on an urban farm, to preparing and eating meals with seniors, to assisting with an all-girls open gym program for Muslim-identified youth. While the total number service-learning hours is on the lower end of ranges recommended (17), it reflects the introductory level of the course and an amount that is realistic for the student population (many of whom work 10–20 h per week to finance their education). The faculty rely on CWL to identify community partners that benefit from engagement of volunteers in short-term experiences. For those organizations interested in a St. Catherine student presence for more than 3 weeks, CWL places students from other service-learning courses at the same organization on alternating weeks, resulting in consistent St. Catherine's partnership with the organization across the semester.

During the final week of class, students present their community health assessment as a written paper and oral presentation to their classmates. Before service-learning was introduced to the course in fall 2015, students selected a community within the Twin Cities arbitrarily, often resulting in superficial understanding of the community and complications for even introductory-level assessment. Thus, the Public Health program contacted CWL to determine how service-learning might facilitate deeper community engagement and student learning in this course. data suggests the pedagogy is meeting this need. Furthermore, encountering service-learning at the start of the major ensures that all students have a foundational understanding of best practices for community engagement in public health settings, and are prepared for more advanced community-based activity scaffolded throughout the major (i.e., project-based service-learning and a 150 h practicum at a non-profit, government, or public health organization).

## Methodology

In order to assess the effectiveness of service-learning as a tool to advance student outcomes related to 2016 CEPH criteria, we submitted an IRB application to gain access to two extant sources of data from the course: student evaluations of service-learning in Foundations for Public Health, and students' written reflections from the course.

At the end of each semester, CWL collects evaluation data from service-learning students via a Qualtrics survey with items related to students' experience and outcomes. Between fall 2015 and spring 2018, there have been 10 sections of the Foundations of Public Health course taught by six different faculty. In these 10 sections, there were a total of 206 students (average of 21 per class) of which, 84 students (41%) responded to the CWL evaluation survey. After removing student identifiers from the data, we examined student responses to four survey items contributing to assessment of the extent to which service-learning advances achievement of the two CEPH criteria selected.

In Foundations of Public Health, students complete a reflection assignment as part of their final Community Health Assessment project which includes addressing how service learning contributed to their educational experience in the course. For this study, the researchers accessed a total of 154 reflections submitted from nine sections of the course between fall 2015 and spring 2018. Reflections were coded for text supporting student achievement of CEPH criteria through service-learning. The researchers also noted exemplars to demonstrate the optimal student outcomes when using this model (Tables 1, 2).

**Table 1:** Frequency and exemplar quotes of socioeconomic, behavioral, biological, environmental, and other factors that impact human health and contribute to health disparities as described in student reflections from *Foundations of Public Health* (Fall 2015–Spring 2018).

**Factor**	**Frequency (%)**	**Exemplars**
Social support and sense of community or belonging	34 (22.1%)	“*…was all about creating a non judgmental environment for people in stressful situation to be apart of so they could experience what it feels like to have a positive community to belong to…If we are able to attempt to understand where people are coming from and how someones life experience can shape everything that they participate in life, it will become easier to provide help for people in disadvantaged situations.”* “*Some elderly do not have a family, so they need a company. They are very happy to see volunteers to come, so they can have a good time to share about life experiences and stories. Because of the needs of care and support, I make sure that they are happy and comfort when I am with them.”*
Food access	30 (19.5%)	“*…lessens the problem of food inequity. When [sic] great thing that [the organization] does is that the vegetables and fruits are not weighted like other foods. Each family can get as many fruits and vegetables as they want…”* “*This farm gives its people…a chance at healthy food. In other neighborhoods, people don't get much fruits and vegetables…This place has shown that with people working together, they can grow more than fresh produce, but they can grow a community.”*
Language and culture	26 (16.9%)	“*It was designed in a way that approached language through lessons that delivered specific language education encapsulated into information in the following areas: public health, safety, personal health and nutrition…This type of education and community commitment can breakdown barriers in access to health care, which is a key social determinant in health outcomes.”* “*Language barrier puts limitations on an individual not living in their native land. It can cause misunderstanding can result in them being taken advantage of.”*
Housing/shelter	22 (14.3%)	“*…those without homes don't have a lot of resources, and the resources they do have are lacking resources themselves…they carry a heavy stigma…”* “*Jane Doe had not eaten all day, and she passed out in line while waiting to get in…Sitting down with talking with her, I found out that she was living in a hotel; Jane Doe was told she couldn't stay at the hotel anymore due to lack of payment…”*
Education	20 (13.0%)	“*I feel like the areas of social justice that need to be worked on in academic situations are above my control. Things like admission rates for minorities and affordable education for all students are all things I cannot change by myself.”* “*It seemed to me that we were able to help the kids who really needed it in order to close the education gap.”*
Age and ability	17 (11.0%)	“*The class is designed for all levels and focuses on engaging you as a whole and interacting with one another. I think that's a huge part of healing and coping with a chronic and debilitating illness.”* “*It gave me new perspective on the population with developmental and intellectual disability and understanding of how this social group are excluded from the large population.”*
Incarceration	14 (9.1%)	“*Where many women are in prison are there due to some kind/type of social injustice. Most women that are in prison come from median to low income families/communities where violence is higher and the chances to enter a circle of violence is even higher.”* “*After finding out that the statistic of women in prison is due to domestic violence, and misdemeanor crimes such as theft, and drug possession, I came to realize that many of the women were there because of self-defense, or were trying to make a living within the circumstances they were in.”*
Gender	14 (9.1%)	“*This program is for young Muslim and Somali girls. It empowers and unites them through sportsmanship…I felt I could relate to it…I was once a young girl with plenty of insecurities…Playing sports was one of the few things that established confidence in me as a child.”* “*These women's stories showed me that sometimes you do not realize you are in a violent relationship until after you leave and look back. They hurt themselves without ever realizing that they were hurting.”*
Employment	9 (5.8%)	“*The business also helps them gain some work experience which ultimately helps them in their future…”* “*…in service-learning by promoting the welfare of individuals in poverty (all pertaining to immigrant/refugee populations)…to increase the job opportunities available to these individuals, as well as general necessary knowledge…These services are necessary for the students to gain independence within the community and provide opportunities for their families.”*
Violence/trauma	9 (5.8%)	“*…opened my eyes into the world of sexual violence and the issues that arise from this injustice.”* “*I learned so much about what sexual violence was and how many people it really affected. I also learned that it wasn't just physical, it was also mental and emotional…”*
Access to medical care	8 (5.2%)	“*…Americans must be able to access healthcare as a right. It is almost impossible to get the healthcare you need without a state or any type of picture ID.”* “*It seemed like he was a young kid stuck in a grown man's body. It made me realize he might not have been getting the health resources he needed.”*
Race	8 (5.2%)	“*To think that some people are at a disadvantage because of their race, the color of their skin hurts. I realized how we all say we have come a long way and how racism isn't prevalent yet some people are experiencing racism and are directly affected by that fact.”* “*They unite communities by community services and fight racism through training and institutional change.”*
Environment	6 (3.9%)	“*Having green space and safe environment supports people getting outside and becoming more active, getting to know other people and building a community that keeps an eye out and improves the overall safety of the community.”*
Income	2 (1.3%)	“*The programs aims to reduce the causes of poverty, rather than just financial assistance.”*
Genetics	1 (0.6%)	“*Genetic risk factors and deep brain stimulation are of the most interest to me because of how many opportunities for understanding the disease better they both offer…”*
None	10 (6.5%)	N/A

**Table 2:** Frequency and exemplar quotes of themes and sub-themes that demonstrate the support of experiential learning to didactic public health education in *Foundations of Public Health* (Fall 2015–Spring 2018).

**Theme**	**Sub-themes**	**Frequency (%)**	**Exemplars**
Theme 1: Foundational concepts of health, public health, and health systems	Social determinants of health	59 (38.3%)	“*The more educated they are, the more they may learn…and will be able to find resources for better health.”* “*This type of education and community commitment can breakdown barriers in access to healthcare, which is a social determinant in health outcomes.”* “*The homeless community needs support, education, healthcare and jobs.”* “*…people from the shelter have lack access to get things that they need because they don't get the opportunity to learn how to get a job, or have nice cloth so that they can get hire.”*
Theme 1 cont.	Community-based interventions	19 (12.3%)	“*…also caters to the needs of the community.”* “*…an essential part of some of the children's education, and well-being of the community in general.”* “*…locals will have the opportunity to receive fresh crops by participating in farm activities.”*
Theme 1 cont.	Definitions and aspects of health	12 (7.8%)	“*When people feel they have a community that fulfills their social needs, they can put more effort into other aspects of themselves, often in the case of Parkinson's disease, this means keeping symptoms at a minimum.”* “*…provides an opportunity to alleviate pieces of the emotional strain.”*
Theme 1 cont.	Health disparities	5 (3.2%)	“*…mentioned systemic racism a the reason why they found themselves in their current state and some of the men experience discrimination and feel as if their voices are unheard.”*
Theme 1 cont.	Health systems and access to healthcare	5 (3.2%)	“*…impossible to get the healthcare you need without a state or any type of picture ID.”*
Theme 1 cont.	Social-ecological framework	4 (2.6%)	“* I think this is true in many of the circles we belong to…we are starting to understand that people need more than just safe housing, clean water, and access to food, they also need positive community and green space…”*
Theme 2: Social justice		64 (41.6%)	“*Service plays a huge role in social justice; you are taking action and as a result, moving toward a solution/something better.”* “*…making everyone feel equal in every way they could possible…it was hard to tell who was a volunteer and who was there because they were in need.”* “*I think that educating people or ourselves in general is the first and most important step in social justice.”* “*Social justice means that the minimal income, housing, employment, education, and healthcare should be seen as a fundamental right.”* “*…part of social justice because we are fighting for equal treatment…we support their human rights and we continue to push for the fair allocation of community resources.”*
Theme 3: Beginning-level professional attitudes, behavior, and communication	Advocacy and awareness	33 (21.4%)	“*This experience gave me a stronger sense of connection to…the disabled community It strengthened my desire to support and advocate for these communities.”* “*We can advocate for the most vulnerable populations and do our best to get them the services they need.”* “*I can use social media to spread the word about good organizations…”*
Theme 3 cont.	Communication skills	12 (7.8%)	“*It got easier talking and helping people…”* “*I have never done a casual interview before to people that I had just met. This made me more comfortable speaking with others.”*
Theme 3 cont.	Future public health roles	11 (7.1%)	“*Knowing that I have a connection to a place that I could get an internship or job at someday is a really positive take away that I received from this experience.”* “*…this experience prepares me and gives me the idea of what i could do in the future to help families and communities.”*
Theme 3 cont.	Teamwork	5 (3.2%)	“*We were part of a team. We all served a purpose there and did our best to achieve the mission of the organization…”*
Theme 3 cont.	Accountability	4 (2.6%)	“*…go for the full time that we are scheduled for; not arriving late nor leaving early. I personally feel that that shows disrespect to the site…carry out what you said you were going to do.”*
Theme 3 cont.	Problem-solving	2 (1.3%)	“*[people] are in the wrong area of public health if they cannot get their feet a little wet in real problems situations…my overall experience was great and look forward to working in Public Health!”*
None		10 (6.5%)	N/A

## Findings

In reviewing CWL evaluation data, students from Foundations in Public Health valued their service-learning experiences. Eighty-six percent of students reported that they would recommend service-learning to other students, 9% reported they might recommend, and only 5% reported they would not recommend service-learning. Of the 5%, most did not express concerns about the value of the pedagogy, but instead, their ability to meet the requirement due to time, work, or life constraints. As multiple factors can impact students' experience, we view this data point as a positive indicator for use of service-learning in public health introductory courses. In addition to students' appreciation for the experience, the following data supports service-learning as an effective tool for meeting CEPH criteria.

**Criterion 1: Students should have learning experiences that address the “socioeconomic, behavioral, biological, environmental, and other factors that impact human health and contribute to health disparities” (Foundational Domains)**

The foundational domains outlined in the CEPH criteria are heavily supported by the inclusion of service-learning in the Foundations of Public Health course. On the CWL evaluation survey, 88% of students agreed or strongly agreed that service-learning increased their understanding of diverse and global perspectives, which equips students with new lenses for understanding health and health behaviors. Additionally, 88% of students agreed or strongly agreed that service-learning increased their understanding of social justice, which aids students in identifying and addressing health disparities.

The contributions of socioeconomic, biological, behavioral, environmental, and other factors to the persistence and–ideally–elimination of health disparities were articulated by students in their end of semester reflections for the Foundations of Public Health course (Table 1). The most commonly reported factors mentioned in student reflections were focused on social support and a sense of community or belonging, food access, language and culture, housing/shelter, education, and age/ability (Table 1). The direct links to service-learning sites for many of these factors is often straightforward. For example, students were placed at organizations where there were food shelves and community gardens (food access), they tutored English-language learners (language and culture), and distributed hygiene packs and bedding at homeless shelters (housing/shelter). However, the students most frequently reflected on the role of their service-learning organization in contributing to a sense of community and belonging for the individuals it served. This factor was present across all semesters, suggesting that, regardless of community placement, students recognized the importance of social supports to individual and community health. This was most profoundly stated by one student who wrote that her organization

“was all about creating a non-judgmental environment for people in stressful situation to be apart [sic] of so they could experience what it feels like to have a positive community to belong to…If we are able to attempt to understand where people are coming from and how someones [sic] life experience can shape everything that they participate in life, it will become easier to provide help for people in disadvantaged situations.”

In spite of some grammatical errors (not uncommon as many of our students are non-native English speakers), nearly all reflection papers mentioned at least one factor related to human health and health disparities (93.6%, *n* = 145).

**Criterion 2: Students should be involved in experiential learning activity to support didactic education (Cumulative and Experiential Activities)**

Another undergraduate CEPH criterion states that students should be involved in experiential learning activities that support didactic education. According to evaluation data from students who completed service-learning as part of Foundations in Public Health between fall 2015 and spring 2018, students found service-learning to be a valuable part of their course learning. Of the 198 students who completed service-learning in the Foundations of Public Health course between fall 2015 and spring 2018, 84 responded to the evaluation survey from the CWL, for a response rate of 42%. Eighty-two percent of students agreed/strongly agreed that service-learning enhanced what they learned in the course, and 86% of students agreed/strongly agreed that course activities and assignments connected their service-learning to their classroom learning.

While there are several specific learning objectives in Foundations of Public Health, the learning objectives can be grouped into three primary themes: (1) foundational concepts of health, public health, and health systems; (2) social justice implications of public health; (3) beginning level professional attitudes, behavior, and communication. Through student reflections, it was clear that these didactic elements were supported through service learning in the course (Table 2). Only ten (6.5%) students did not mention at least one of these learning objectives in their reflections. Within the first theme, examples included in the reflections focused on describing the social determinants of health, health disparities, definitions and aspects of health, health systems and access to healthcare, the social-ecological framework, and community-based interventions. Succinctly put, one student described social determinants in the homeless community as, “*The homeless community needs support, education, healthcare and jobs.”*

The most frequent theme across the student reflections focused on social justice in the work of the community organization. Elements of social justice were identified in 64 (41.6%) of student reflections. One student wrote in her reflection, “*Service plays a huge role in social justice; you are taking action and as a result, moving toward a solution/something better.”*

Notably, students also described specific skills and interests at their sites that would serve them well in future public health work including communication skills, advocacy, and problem-solving, and future public health roles. Many students expressed a desire to share their new knowledge with others. “*This experience gave me a stronger sense of connection to…the disabled community It strengthened my desire to support and advocate for these communities.”*

One aspect of St. Catherine University's Catholic mission is the importance of reflection, and most importantly, self-reflection. Although not evaluated as a learning-outcome, many students explored relationships between their service-learning site and their own lived experiences in their reflection papers without prompting. In our undergraduate public health program, two-thirds of our traditional students and one-third of students in our adult learner program identify as students of color. More than one in four undergraduate public health students are multilingual. Throughout their reflections, students shared how they had either benefitted from similar service-learning organizations when they were younger, and what barriers they and/or their parents faced as new immigrants to the United States:

“I learned English when I was 10 years old and have experienced the struggle of learning a language and how it feels to have a language barrier…Language barrier puts limitations on an individual not living in their native land. It can cause misunderstanding can result in them being taken advantage of.”

Others mentioned having received food from food shelves because their family did not have enough food, and still others shared their experiences with domestic violence. Many described their appreciation of service-learning as a way to give back to the community.

## Discussion

Data supports the continued use of service-learning in St. Catherine University's undergraduate public health program to meet CEPH student outcomes related to determinants of health and experiential learning. The foundational domains of CEPH accreditation criteria state that students should have learning experiences that address the “socioeconomic, behavioral, biological, environmental, and other factors that impact human health and contribute to health disparities.” Certainly the definitions and examples of these health factors need to be introduced in a classroom setting, and service-learning does not replace the required knowledge acquisition. Rather, service-learning does reinforce concepts discussed in the classroom by provided students with examples of determinants of health as they appear in their community context. These connections were well-articulated by students in their service-learning reflections. We have also found it important to provide students with service-learning in a foundational course so that they are able to better conceptualize the breadth of what constitutes working in public health. This helps to meet the CEPH accreditation criterion related to cumulative and experiential activities that support didactic education. As described in their reflections, students reported demonstrating beginning professional attitudes, behavior, and communication skills in public health. The St. Catherine University Public Health program's application was accepted for accreditation, and we are actively completing the self-study process. Feedback from our CEPH consultation visit was overwhelmingly positive with respect to the service-learning elements of our undergraduate program.

As we recommend service-learning as a pedagogy for undergraduate public health, we recognize it may not be feasible or beneficial for all programs; as Enos and Trope (18) observe, while “it is possible to incorporate [service-learning] in any discipline,” service-learning is “not destined to be used in every course” (p. 159). Furthermore, we do not advocate that our exact model be replicated. Rather, we encourage departments to consider how this pedagogy might be applied to their program in a way that that reflects the unique needs of their student, faculty, and community stakeholders. Understanding best practices of community engagement along with the history, power and privilege at play in university-community partnerships is key to successful service-learning design and implementation. As undergraduate public health programs contemplate utilizing service-learning, the following lessons from our own experience may also be helpful.

### Timeline

Service-learning requires intentionality in design and relationship development, which take time. We recommend starting to plan at least 1 year in advance.

### Capacity

If your institution does not have a community engagement office able to cultivate community partnerships and advise on service-learning, consider whether faculty workload allows for the additional time required for relationship development with community partner organizations and researching service-learning pedagogy. Because of the commitment to partnerships and pedagogy, as well as to ensure continuity of messaging around the importance of service-learning to our public health program, we have found that primary instructional faculty are best-suited to incorporate service-learning into their courses.

### Design and Implementation

Prior to considering your course or program design, we recommend reaching out to colleagues on campus with service-learning experience; consulting literature such as *Service-learning Essentials* (19) or *The Cambridge Handbook of Service Learning and Community Engagement* (13); and connecting with your regional Campus Compact office. (20) draws a distinction between service-learning that is “placed in the syllabus as something that is supplemental and periodic,” and service-learning that acts as the “experiential base of the course with a full array of activities and exercises to support it and to amplify course material” (p. 33). To achieve this latter model in a way that simultaneously seeks reciprocity for community partners, consult with extant theory and practice-based resources available; sharing on lessons learned can be particularly valuable.

As faculty approach implementation, it is important to note that service-learning may require new or unfamiliar approaches to instruction. Critical reflection should be integrated throughout the course and can occur in different formats, such as speaking, writing and classroom activities (19). We cannot stress enough the importance of thoughtful reflection design, and there is empirical evidence that the “degree of written and oral reflection influence[s] the effectiveness of service learning” [(21), p. 224]. Although journaling and written reflection are popular methods, “other classroom teaching and learning activities can help students make connections as well” [(22), p. 51].

The pedagogy is also not conducive to exclusively lecture-style courses, and “challenges faculty to reconsider their constructions of control and authority in the classroom and beyond” [(18), p. 159]. Bringle et al. (14) describe this as a “paradigm shift in higher education because it heightens the role that students can assume as constructors of knowledge” (p. 7). Finally, service-learning may require professional development coaching from faculty as students work on communication and relationship-development skills in environments outside of the classroom. As one undergraduate public health program reported from their model of community-engaged learning in a capstone course, “faculty serve not only as instructors and mentors but also as conveners, resources, coaches, mediators, managers, assessors, and motivators” [(23), p. 97].

### Evaluation

As with most curriculum, refining service-learning in a course is an ongoing process. Our evaluation data from students and community partners informs course adjustments and is one more avenue for understanding and maintaining relationships. While we were able to retroactively draw from our evaluation data for identifying the meeting of CEPH criteria, programs just beginning to consider service-learning have the opportunity to design evaluation in a way that more explicitly assesses how service-learning advances student outcomes required by accreditation.

## Conclusion

In completing this study, our findings support service-learning as an effective pedagogical model for introductory undergraduate public health coursework. In St. Catherine University's *Foundations in Public Health* course, service-learning facilitates achievement of student learning outcomes related to understanding the “socioeconomic, behavioral, biological, environmental, and other factors that impact human health and contribute to health disparities” (CEPH Foundational Domains). Furthermore, by nature of its design, the pedagogy provides students with experiential learning that enhances their didactic learning (CEPH Cumulative and Experiential Activities requirement). Although the use of extant data sources limited the opportunity to compare achievement of the first criteria with a control group of students without service-learning, our data suggests that students with service-learning achieve learning outcomes desired while also meeting the second CEPH criteria around experiential learning (something that would not happen without service-learning). The original motivation for including service-learning in St. Catherine's *Foundations of Public Health* course was informed by student and community partner interest in combination with the University's value for social justice and community engagement. However, we have come to realize the pedagogy offers an additional benefit of meeting multiple CEPH criteria essential to the success and accreditation of our undergraduate public health major—something other undergraduate public health programs may want to consider as well.

## Data Availability

The raw data supporting the conclusions of this manuscript will be made available by the authors, without undue reservation, to any qualified researcher.

## Ethics Statement

This study was granted exempt level approval from the St. Catherine University Institutional Review Board because it used previously collected, de-identified, evaluation data.

## Author Contributions

MM and ED both contributed to the design and implementation of the research, to the analysis of the results, and to the writing of the manuscript.

### Conflict of Interest Statement

The authors declare that the research was conducted in the absence of any commercial or financial relationships that could be construed as a potential conflict of interest.
