# Supplementary material for: Service-Learning as a Practical Introduction to Undergraduate Public Health: Benefits for Student Outcomes and Accreditation
Source: Front Public Health. 2019 Apr 2;7:63. doi: 10.3389/fpubh.2019.00063 (PMC6454065; doi:10.3389/fpubh.2019.00063)
Supplement: Supplementary file 1 [file Table_1.docx]

HLTH 2050 - Foundations of Public Health: A Social Justice Perspective

Community Health Assessment

**Grading:**

This assignment will be done in groups of 3 or 4 as determined by your service learning sites. Students will be graded on the final product as a group, meaning you will all receive the same grade. The presentation is worth 5% of your final grade in the course, and the group paper is worth 10% of your final grade in the course. There is also 2.5 % of your overall grade in the course under the “Participation” category that determined by feedback from your group members on your contributions to the project. An additional 10% is your service-learning attendance and individual reflection paper.

**Purpose**:

The purpose of this assignment is to practice conducting a community health assessment by focusing on the strengths and assets of a community as well as stressors (where the community lacks resources). By incorporating a Community Work and Learning experience, students will better understand and relate to the community members. Students will also continue to build on their writing skills by combining quantitative and qualitative data to describe their community of choice. Students will be asked to view their communities through the lens of social determinants of health. The topic/community of the paper and presentation will be a particular neighborhood in Minneapolis or Saint Paul in which the students’ service learning site was located.

**Students will:**

1. Locate and interpret quantitative data about the social determinants of health in a community.
2. Explore a new community via a “walking tour” and summarize the strengths and needs of the community.
3. Engage in conversation with community members through key informant interviews.
4. Participate in the community through a Community, Work, and Learning experience.
5. Demonstrate the ability to read, think, and write critically at an introductory level in the field of Public Health, and cite sources correctly.
6. Develop skills as public health professionals in group work, professional presentations, and the creation of a technical report.

**Guidelines for Community Health Assessment Paper**

**Page 0: Title Page**

- Name of community
- Name of CWL site
- Group number
- Names of group members
- Date

**Page 1: Community Definition and Description**

- In which city is the community located?
- What are the boundaries of the community? (river, certain streets, zip code, etc.)
- Any interesting historical facts about how the community came to be and why it exists
- Is the neighborhood near anything iconic? Airport, college, transit center, etc.

**Pages 2-4: Quantitative Data and Statistics (3-5 paragraphs) –** *Remember to analyze the data you have collected. Write evidence-based sentences about what these data tell you about the community being studied.*

Example of types of data and charts you may present (see “Community Profile”): <http://lee.floridahealth.gov/programs-and-services/community-health-planning-and-statistics/chip-cha/_documents/FDOH-Lee-CHA-CHIP-September-2014-Edition.pdf>

Data:

- Demographic information (age distribution, income distribution, ethnicity)
- Housing availability (Rental vacancy rates, Monthly rent, Homeownership rates, Median household price)
- Crime statistics
- Morbidity and Mortality rates
- Transportation resources, commute time
- Healthcare facilities
- Parks and bike trails

Analysis:

- Are these data consistent over time or has the community transitioned in wealth or ethnicity or otherwise?
- How might these data influence the health status of community members?
- What are the primary causes of morbidity and mortality? How is that affected by the demographic information or other quantitative data?

**Pages 5-7: Walking tour (3-5 paragraphs)–** *Remember to analyze the data you have collected. Write evidence-based sentences about what your observations tell you about the community being studied.*

More information about walking tour: <http://ctb.ku.edu/en/table-of-contents/assessment/assessing-community-needs-and-resources/windshield-walking-surveys/main>

Example from Wisconsin students: <https://www.youtube.com/watch?v=TGXMoQjXbUk>

Data:

- Who do you see when you drive through the community?
- What do you see? New houses? New industry? Abandoned buildings? Broken windows? Poorly maintained buildings? Gardens? Barriers or facilitators to movement? Public transportation?
- What types of services are available? Full-service groceries? Convenience-type stores? Pharmacies? Other Stores? Transportation? Schools? Recreation?
- Visit the community at different times of day.  Is this a place where the activity ends before dark or starts after dark?
- Where are essential services located?
- Where are people congregating?  Where are there no people?
- Where are the roads, sidewalks and parks?
- How does the community feel--prosperous, depressed, and/or safe?

Analysis:

- How does the walking tour align with quantitative data that you found? Do you see people of the most common ethnicity? Does it seem to fit the income data? Etc.
- How might what you see impact the health of the population in that community?
- What services seem to provide the largest benefit for the community? How would that influence healthy behaviors? What lack of services prevents healthier behaviors from taking place?
- How might the “feel” of the community influence community members’ health?

**Pages 8-10: Key Informant Interviews (2-4 paragraphs)**

Possible people to interview:

- Business persons
- Community center workers
- Residents (waiting for buses, at a coffee shop, in the library)
- Educators
- Council person(s)

Data (possible interview questions):

- What would you say are some of this community's strengths?
- What are some of the gifts and talents of the people here?
- What is the community's greatest source of pride?
- Who are the people in the community who take care of others when it is not part of their jobs? For example, who makes sure that children are safe; who makes sure that families have food?
- What groups, clubs, or associations in the community make a difference in the well-being of the community?
- To what extent do people in this community know their neighbor?
- What are the most significant human service issues or problems facing your community at the present time?
- In your opinion, what are the most significant unmet needs in your community at the present time?
- What are the greatest challenges or barriers to the community residents and agencies of your community?

Analysis:

- How do the two interviews compare? What are similar themes? How or why might these two individuals view the community differently?
- What additional information did these interviews provide you with that the data and walking tour could not?
- What did the informants say that was similar to your data? For example, did they reference a particular ethnic group as being a large portion of the population? If so, does the demographic data show that?
- How does the “biggest challenge”, as identified by the informant, affect community members’ health?

**Page 11: Community Work and Learning Experience (1-3 paragraphs)**

Site name and type of experience

- Type of experience (food shelf, shelter, educational)
- Type of residents (homeless, adult, children)
- How did this experience compare with the information gathered during the key informant interviews – strengthen the information gathered or seem as at odds with it?

**Page 12: Conclusions**

- 1-2 sentences about definition and description
- 1-2 sentences about general demographics or other quantitative data of interest
- 1-2 sentences about walking tour
- 1-2 sentences about informant interviews
- 1-2 sentences about the future of this community and its opportunities and challenges in improving its members’ health

**Pages 13-14+: References and Appendices**

- Use APA format for your references
- If you have any charts/tables/graphics/maps you want us to see, please include them as appendices.
- Title your appendices

**Rubric for Community Health Assessment Presentation**

|  | \|  \|  \|  \|  \| \| --- \| --- \| --- \| --- \| \| **Criteria** \| **Excellent** \| **Good** \| **Adequate** \| \| **Content** \| 5 points  Content is appropriate for the assignment; it clearly describes the community or neighborhood. Explains its strengths (assets) and weaknesses using appropriate quantitative data and interview data. Provides critical analysis of the community. Concludes with recommendations for this community and its opportunities and challenges for improving its community members’ health. \| 4 points  Provides good data but lacks some critical analysis and/or the content is unbalanced with too much data in one area and too little in another. \| 3 points  Lacks some key data requirements; may lack some critical thinking and/or is unbalanced in its description of the community. \| \| **Delivery** \| 4 points  Each group member speaks for about the same amount of time. Speakers are confident, poised and enthusiastic. Volume and rate varies to add emphasis and interest. Pronunciation and enunciation are very clear. Style is conversational, with appropriate use of notes. Speaker does not read the slides word for word. Speaker has good eye contact. \| 3 points  Most members have good delivery but one or more are very subdued; may read slides word for word; may have limited eye contact with audience. May have poor transition between speakers. \| 2 points  Two or more speakers have weak delivery; there is poor transition between speakers. \| \| **Organization** \| 2 points  Message is clearly organized. Speakers use organizational aids: announcing topic, preview discussion, transitions, and summarizes. \| 1 point    Message is understandable. Sequence is not clear or difficult to follow \| 0.5 points    Difficult to understand due to disorganization \| \| **Creativity** \| 2 points  Original presentation of material. Captures the audience’s attention. A variety of supporting material is used to engage audience \| 1 point  Some originality apparent; Could use more variety. \| 0.5 points  Repetitive with little or no variation; insufficient use of materials \| \| **Timing** \| 2 points  Stays within 1-minute of allotted time (15-20 minutes). Not less than 14 minutes; not more than 21 minutes. \| 1 point  Two minutes under or over allotted time. \| 0.5 points  Three minutes or more over allotted time. \| |
| --- | --- | --- | --- | --- | --- | --- | --- | --- | --- | --- | --- | --- | --- | --- | --- | --- | --- | --- | --- | --- | --- | --- | --- | --- | --- | --- | --- | --- | --- |

**Outline for Community Health Assessment Presentation*:**

Slide 1 – Title, Neighborhood, Group Member Names

Slide 2 – Outline of your presentation

Slide 3 – Map of Neighborhood

Slide 4- Geographic and Historical Context

Slides 5 – 10 – Data, Charts, Graphs – Quantitative Information

How do these data influence health in that community?

Slides 11-19 – Walking Tour (Show lots of pictures!)

Slides 20-25 – Key Informant Interviews

Use quotes from interviews (and photos, if possible)

Slides 26-28 – Community Work and Learning Experience

Slides 29-32 - Other exciting information about the community, opportunities for public health interventions in the community, things that surprised you and your group

Slide 33 – Conclusion about the strengths and needs of the community

*Note, this is just a guideline, please modify your group’s presentation as you see fit.

**Resources to get you started:**

*United Way Neighborhood Profiles:*


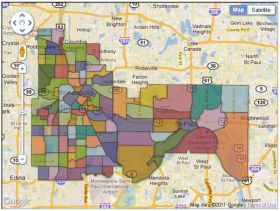
 [*http://www.mncompass.org/profiles/neighborhoods/minneapolis-saint-paul*](http://www.mncompass.org/profiles/neighborhoods/minneapolis-saint-paul)

*Demographic Information Sources:*

Minnesota State Demographic Center: <http://mn.gov/admin/demography/>

United States Census: <http://www.census.gov>

City of Minneapolis: <http://www.ci.minneapolis.mn.us/>

CityData.com: <http://www.city-data.com/zips/55408.html#ixzz2jEJ6LRQ0>

Zip-Codes.com:<http://www.zip-codes.com/zip-code/55408/zip-code-55408.asp>

Movoto.com*:* <http://www.movoto.com/neighborhood/mn/minneapolis/55408.htm>

Neighborhoodlink.com: <http://www.neighborhoodlink.com/zip/55408#overview>

*Providers of health services*:

Minnesota Department of Health: <http://www.health.state.mn.us/divs/fpc/directory/providerselect.cfm>

*Transportation, Parks and Recreation:*

Metro Transit: <https://www.metrotransit.org/>

Car to Go: <https://www.car2go.com/en/minneapolis/>

Minneapolis Parks and Recreation: <https://www.minneapolisparks.org/>

Saint Paul Parks and Recreation: <http://www.stpaul.gov/index.aspx?nid=243>
